# Latent profiles and predictors of barriers to care in Swiss children and adolescents with rare diseases

**DOI:** 10.1093/jpepsy/jsae076

**Published:** 2024-09-24

**Authors:** Susanne Wehrli, Matthias R Baumgartner, Andrew A Dwyer, Markus A Landolt

**Affiliations:** Department of Psychosomatics and Psychiatry, University Children’s Hospital, University of Zurich, Zurich, Switzerland; Division of Child and Adolescent Health Psychology, Department of Psychology, University of Zurich, Zurich, Switzerland; Division of Metabolism, University Children’s Hospital Zurich, University of Zurich, Zurich, Switzerland; Boston College, William F. Connell School of Nursing, Boston, MA, United States; P50 Massachusetts General Hospital—Harvard Center for Reproductive Medicine Boston, Boston, MA, United States; Department of Psychosomatics and Psychiatry, University Children’s Hospital, University of Zurich, Zurich, Switzerland; Division of Child and Adolescent Health Psychology, Department of Psychology, University of Zurich, Zurich, Switzerland

**Keywords:** rare disease, chronic illness, access to care, healthcare, barriers to care, child health

## Abstract

**Objective:**

Children and adolescents with rare diseases face significant barriers when accessing healthcare. We aimed to assess and predict these barriers and investigate associations with health-related quality of life (HRQoL).

**Method:**

We conducted a cross-sectional survey of Swiss parents (*N *=* *189) of children with rare diseases including the Barriers to Care Questionnaire (BCQ), containing six barriers and the Pediatric Quality of Life Inventory (PedsQL). Latent profile analysis (LPA) was used to uncover distinct classes, which were compared using chi-square tests and Mann–Whitney *U* tests. Relevant medical and sociodemographic class predictors were identified using Elastic Net regression, followed by regression analysis to investigate their role in predicting barriers to care and examine the effects of these classes on HRQoL.

**Results:**

Two distinct groups were identified, a higher barriers class (59%) and a lower barriers class (41%). In the higher barriers class, participants showed elevated scores across all subscales and specifically on pragmatics and expectations. More barriers to care were linked to a nonstable disease course (OR = 2.27, *p *=* *.002) and a diagnosis after the age of 3 months (OR = 2.17, *p *=* *.006). Individuals in the higher barriers class exhibited more psychological comorbidities (*p *=* *.044), congenital malformations/deformations/chromosomal abnormalities (*p*=.042), and medical misdiagnoses (*p *=* *.006). Children in the higher barriers class had significantly lower PedsQL scores compared to the lower barriers class (*p* <.05).

**Conclusion:**

This study highlights the need for comprehensive assessment of barriers to pediatric care in rare diseases, offering potential entry points for targeted interventions.

Rare diseases are often chronic, genetic, incurable, and life-limiting conditions (Nguengang Wakap et al., 2020). Despite their classification as “rare,” the collective prevalence of these diseases is substantial. Recent estimates suggest that 3.5%–5.9% of the world’s population has been diagnosed with a rare disease (EURORDIS, 2021; Nguengang Wakap et al., 2020). The definition of what makes a disease rare varies from country to country and from organization to organization, resulting in numerous definitions (Richter et al., 2015). Approximately 80% of all rare diseases are genetic in origin and about 75% of rare diseases affect children (Dodge et al., 2011). Cystic fibrosis, for example, has a prevalence of 5 in 10,000 in Europe. This complex, life-limiting genetic disease has seen dramatic improvements in survival rates, reflected in increased life expectancy (Graf von der Schulenburg & Frank, 2015). In contrast, Batten disease is a childhood neurodegenerative disorder with a much lower prevalence of around 1 in 100,000 births. There are few treatments and early death is almost certain (Johnson et al., 2019). These two examples illustrate that rare diseases vary not only in their prevalence, but also in the different impact they can have on the lives of the families affected.

Families facing rare diseases often encounter a variety of challenges (Pelentsov et al., 2015). Parents of affected children experience profound changes in their lives that may require adjustments in work, income, caregiving, and family dynamics. It is therefore not surprising that parents frequently report feelings of isolation, anxiety, and frustration (Pelentsov et al., 2015). Given the early onset and chronic nature of most rare diseases, families often have ongoing interactions with the healthcare system. Accessing healthcare can be quite complicated, but efficient access to care is related to better health outcomes for children with chronic conditions and is accordingly essential (Boulet et al., 2010). Potential reasons for challenges in access to healthcare have been identified as limited access to specialists due to long waiting times and low numbers of specialists, difficulties in accessing insurance payments, and scarce information about the disease, its care, and treatment (Pasquini et al., 2021; Zurynski et al., 2017). Caring for a child with a rare disease often results in a significant treatment burden due to the demands of the healthcare system on the caregiver and the patient, such as care coordination and medical appointments (Hannan et al., 2021; Zurynski et al., 2017). Shared burdens have also been reported for more common chronic diseases, such as type 1 diabetes or asthma, and include conflict between the disease and personal life, stigma from the surrounding community, and reduced mental health (Epping-Jordan et al., 2004; Moussavi et al., 2007). However, there are likely to be additional challenges related to the low prevalence of rare diseases that may be more specific to this group, which is particularly visible in healthcare in terms of patient and provider knowledge, limited access to specialists, and increased waiting times (Uhlenbusch et al., 2019b).

Previous research has uncovered disparities in access to care among children and adolescents with rare diseases, yet it is critical to understand the barriers contributing to inadequate access. Barriers to care, as listed below, are defined as socio-behavioral processes that adversely impact families’ interactions with the healthcare system, making access challenging (Seid et al., 2004). Barriers to care therefore go beyond the concept of access and provide insights into the broader process that goes beyond being a mere indicator of poor healthcare access. These barriers encompass pragmatic factors (such as logistical and cost-related issues), skills (the ability to interact effectively with the healthcare system), expectations (anticipation of substandard care), marginalization (internalizing negative healthcare experiences), and the knowledge and beliefs component (belief in conventional medicine) (Seid et al., 2004). Previous research has demonstrated that these barriers impede access for families with children and adolescents dealing with chronic illnesses (Jacob et al., 2016). For example, a study of youth with type 1 diabetes found that the three most common barriers were cost, communication, and obtaining needed information (Valenzuela et al., 2014). Similarly, for children with asthma, the top three barriers were knowledge and beliefs, stigma, and expectations (Seid et al., 2009). Nonetheless, the barriers outlined by Seid et al. (2004) remain largely unexplored within the context of rare pediatric conditions.

In children with rare diseases, the concept of health-related quality of life (HRQoL) has emerged as an important health outcome. HRQoL is a multidimensional construct that captures an individual’s physical, functional, social, and emotional health status (Karimi & Brazier, 2016). Several studies show that children with rare diseases exhibit significantly lower HRQoL compared to healthy controls and norms (Wehrli et al., 2023; Witt et al., 2019). Importantly, previous research has also revealed a link between barriers to care and child health outcomes, including HRQoL. These findings have sparked the potential that addressing and mitigating barriers to care could improve health outcomes (Seid et al., 2009). The model by Seid et al. (2004) underscores the significance of a sequence of critical steps to ensure improved health outcomes. These steps encompass the availability of care, actualized access to care, the delivery of high-quality care, adherence to healthcare provider recommendations, as well as specific predisposing socioeconomic and disease-specific factors. Consequently, barriers to care can potentially act as moderators in the relationship between these steps, potentially diminishing health outcomes such as HRQoL.

Previous research indicates that socioeconomic factors, insurance coverage, and personal circumstances impact healthcare access for rare diseases (Bogart et al., 2022). Unstable disease courses and misdiagnosis pose additional challenges (Birkhäuser et al., 2017). While early diagnosis improves access, these sociodemographic and disease-specific factors are understudied in pediatric rare diseases, highlighting the need for research to alleviate caregiver burden and enhance child health outcomes (Hannan et al., 2021).

##  

### Objectives

The objectives of our study were threefold. First, we sought to gain a deeper understanding of the challenges and barriers to care faced by families dealing with rare pediatric conditions within the context of the Swiss healthcare system. We utilized latent profile analysis (LPA) to identify distinct classes based on the barriers to care questionnaire (BCQ). We postulated that we would uncover at least one class with a higher number of barriers compared to other classes. Due to the exploratory nature of LPA and the lack of prior work on the BCQ in the context of rare diseases, we did not formulate specific hypotheses regarding the number of classes (or their composition).

Second, while various risk and protective factors have been examined in different chronic diseases concerning healthcare access, as listed in the introduction, we introduce a novel integration of barriers to accessing care as well as potential predictors. Given the absence of prior evidence and established theories pertaining to rare pediatric diseases, we opted for a data-driven approach rather than confirmatory hypothesis testing.

In a third and final step, our aim was to investigate whether barriers to care are associated with HRQoL. We hypothesized that classes experiencing more barriers to accessing care would be associated with lower HRQoL compared to classes with less barriers.

## Methods

### Study design and data collection

An anonymous cross-sectional online survey was conducted in Switzerland starting in February 2023 and ending in June 2023, all included participants provided informed consent at the beginning of the survey. Participants were recruited in collaboration with Swiss rare disease patient organizations and with physicians from several Swiss pediatric University Hospitals, namely Bern, Lausanne, and Zurich. Patients were recruited through newsletters, social media, and email from their doctor. Patient organizations forwarded the study via e-mail to their members. Patients did not receive reimbursements. Informed consent was obtained at the start of the online survey.

Parents had to be aged 18 years or older, with children between the ages of 1 and 18 years, living in Switzerland, and be proficient in either German, English, French, or Italian. Additionally, participants were required to have a child diagnosed with a rare disease and be able to recall the child’s disease name. For the purposes of this study, rare diseases were defined according to the criteria established by the European Parliament and the Council of the European Union (2000), which designates a disease as “rare” if it affects fewer than 1 in 2,000 individuals in the European population which is also the definition used in Switzerland (Federal Office of Public Health, 2023). The final dataset consisted of 189 participants.

A total of four participants were excluded from the study because they did not reside in Switzerland, and an additional 17 participants were excluded because the diseases they reported did not meet the rare disease criteria. Also, two participants were excluded because they did not complete the BCQ. Additionally, five participants exhibiting a uniform response pattern across all items on the BCQ, indicative of careless responding, were excluded from the analysis, meaning that they answered all items in exactly the same way (Wood et al., 2017). Furthermore, we assessed individuals for outlier status based on a boxplot criterion, with the intention of excluding them if they displayed outlier status across all BCQ subscales. No participants were excluded as outliers.

### Measures

#### Sociodemographic and illness-specific factors

Parents provided sociodemographic information including their relationship status (in a relationship vs. single), custody arrangement (sole vs. joint custody), as well as their child’s sex, age, nationality (Swiss vs. other), and child’s medical coverage (privately insured vs. general insurance). Parents reported their educational attainment, categorized into low and high levels. Low education included special education attendance or not completing mandatory school education (up to 9 years), aligned with Swiss compulsory education completion within 9–10 years (Swiss Federal Department of Foreign Affairs, 2021). High education comprised upper secondary school, A-levels, technical school, seminar attendance, or university degrees, including University of Applied Sciences and ETH (Federal Institute of Technology Zurich). Therefore, this dichotomization went hand in hand with meaningful milestones in the Swiss education system and compensated for the low power of educational gradients, which were underrepresented in the present sample.

In addition, parents provided information on the child’s disease course (stable vs. unstable), stable was defined as “It is stable; not expected to change very much over time” and unstable as either “It is progressive; expected to get more severe over time,” “It is episodic; there are periods of time when it is stable or gets better followed by periods where it gets worse,” “It is improving; it is expected to get better over time,” and “Unknown.” Further information involved membership/participation in a patient organization, total healthcare utilization during the last 12 months (number of medical visits in the context of inpatient and outpatient setting), child and parent psychiatric diagnoses (yes vs. no), and previous or current counseling or psychological/psychiatric care during the past 12 months (yes vs. no). Parents also answered whether the diagnosis was made before the child reached 3 months of age, which has been previously used as a threshold for early diagnosis (Bar et al., 2017; Molster et al., 2016). Before indicating the number of misdiagnoses, parents were asked if they remembered the exact number of misdiagnoses. If parents answered negatively, they had the option of either selecting “I don’t know” or selecting a range from the following options: 1–2, 3–5, 5–10, or more than 10 misdiagnoses. In the instances where parents selected a range, the median value within that range was used for analysis.

Parental-reported diagnoses were classified according to the International Statistical Classification of Diseases and Related Health Problems 10th revision (ICD-10). This approach was chosen due to the internal validity and standardized framework of the ICD-10 system, which has been shown to be effective in previous studies on rare diseases (Uhlenbusch et al., 2019a). In 11 cases, the reported diagnoses did not align with the ICD-10 framework but were instead categorized according to the ICD-11 classification system (see Supplementary Table 1). A greater number of cases would have required exclusion if the ICD-11 system had been utilized this is why the ICD-10 system was used instead. Furthermore, participants were excluded if the frequency of their disease type in the current sample was less than 10 cases (see Supplementary Table 2). This criterion was applied to mitigate potential problems associated with a low ratio of events per value, which can lead to inaccurate predictions (Pavlou et al., 2015). This approach has previously been adopted in the rare disease context when using disease type as a predictor (Uhlenbusch et al., 2019a). The subsequent LPA was not altered by the presence of the seven excluded cases (see Supplementary Table 3).

#### Barriers to care

The BCQ, developed by Seid et al. (2004) is a 39-item instrument comprising five subscales capturing different barriers to healthcare. This questionnaire was chosen because it is the only validated questionnaire that measures barriers to care in children and adolescents with chronic diseases (Seid et al., 2009). In the present study, the internal consistency reliability (Cronbach’s alpha) for these subscales was as follows: Pragmatic barriers (α = 0.81; 9 items; e.g., “Having to wait too many days for an appointment”), skill barriers (α = 0.80; 8 items; e.g., “Knowing how to make the healthcare system work for you”), expectation barriers (α = 0.90; 7 items; “Worrying that doctors and nurses will not do what is right for your child”), marginalization barriers (α = 0.91; 11 items; “Impatient doctors”), and knowledge and beliefs barriers (α = 0.79; 4 items; “Disagreeing with the doctor’s orders”). The means and *SD*s of the items are shown in Supplementary Table 3. Parents were instructed to rate the occurrence of each barrier over the previous 3 months using a 6-point Likert scale (0=“no problem” to 6=“almost always”). Items are reverse-coded to obtain scores between 0 and 100, with higher scores indicating fewer encountered barriers (Seid et al., 2009). To ensure accurate translation of the questionnaire into German, French, and Italian, two translators first translated the BCQ into the target language, based on which a consensus version was produced, followed by careful application of the back-translation method, following established guidelines for the translation and cultural adaptation process of patient-reported outcome measures (PROs) (Wild et al., 2005).

#### Health-related quality of life

We used the generic version of the Pediatric Quality of Life Inventory 4.0 (PedsQL 4.0) to assess HRQoL. This well-validated, multidimensional instrument is designed for both self- and proxy-reported HRQoL assessment (Varni et al., 2001, 2011). Age-specific versions of the proxy-questionnaire were employed in this study for children aged 1–12 months (infant version), 13–24 months (infant version), 2–4 years, 5–7 years, 8–12 years, and 13–18 years respectively. The Participants used a 5-point Likert scale (0=“never” to 4=“almost always”) to rate their experiences over the past month. Scoring was done following the standard PedsQL scoring protocol (www.pedsql.org) to yield composite scores for physical (13–18 years α = 0.94; 8–12 years α = 0.91; 5–7 α = 0.92; 2–4 α = 0.91; 13–24 months α = 0.90) and psychosocial health (13–18 years α = 0.92; 8–12 years α = 0.92; 5–7 α = 0.87; 2–4 α = 0.75; 13–24 months α = 0.81).

### Statistical analysis

The data analysis was conducted using R Statistics Software (v4.1.2; R Core Team, 2021). To compare classes, chi-square tests and the Mann–Whitney *U* test were employed, as the variables did not follow a normal distribution, confirmed through the Shapiro–Wilk test. Multiple comparisons were accounted for using Bonferroni correction.

Latent profile analysis was performed using the TidyLPA package (Rosenberg et al., 2018) to estimate barrier classes based on the mean scores of BCQ subscales. Several model types were estimated, differing in variances and covariances. Solutions for one to six classes were computed and compared with various fit indices, including entropy, log-likelihood, Akaike Information Criterion (AIC), adjusted Bayesian Information Criterion (aBIC), Bayesian Information Criterion (BIC), and the Consistent Akaike Information Criterion (CAIC), Lo–Mendell–Rubin Likelihood Ratio Test (LMR) and Bootstrapped Likelihood Ratio Test (BLRT) (Ferguson et al., 2020). Lower AIC, aBIC, BIC, and CAIC values indicate better model fit (Masyn, 2013). An entropy value of 0.80 or higher indicates low classification uncertainty. The LMR test and the BLRT assess goodness of fit and model complexity (Lo et al., 2001). After identifying distinct classes through latent profile analysis, we conducted Mann–Whitney *U* to examine differences in BCQ subscales both between and within classes. This approach allowed us to explore the variations in subscale scores among different classes and within each individual class. By comparing subscale scores across classes and within each class separately, we aimed to gain insight into the magnitude of differences observed on the BCQ questionnaire using Cohen’s D.

To investigate associations between risk and protective factors and BCQ class membership, two multiple logistic regression models were employed. The supervised machine learning method Elastic Net regression was used for predictor selection using the caret package (Kuhn, 2020), which is effective in situations involving numerous predictors, multicollinearity, and overfitting (Zou & Hastie, 2005). Elastic Net combines lasso and ridge regression and steers coefficient estimates toward zero for regularization, reducing overfitting. The process included a fivefold cross-validated Elastic Net regression to identify important predictors, followed by multiple logistic regression models. Logistic regression coefficients were presented as odds ratios (ORs).

PedsQL summary scores were z-transformed to compare age-specific versions. Multiple regression was used to determine if class membership was predictive of PedsQL physical and psychosocial summary scores.

## Results

### Participant characteristics

Descriptive statistics of the sample are provided in Table 1. On average, children were aged 7.6 years, with the majority being male. Approximately half of the sample received their diagnosis within the first 3 months of life and exhibited a stable disease course. The most commonly reported disease types were congenital malformations, deformations, and chromosomal abnormalities.

**Table 1.** Descriptive statistics of sample (*N* = 189).

	*Sample (N = 189)*
**Sociodemographic factors**	
Child age, *M* (*SD*), *Range*	7.57 (4.51), 1–17
Child gender female, *n* (%)	77 (40.74)
Education high^a^, *n* (%)	
Mother	165 (87.30)
Father	156 (82.54)
Medical cost coverage private, *n* (%)	30 (15.87)
In a relationship or married, *n* (%)	174 (92.06)
Child Nationality, *n* (%)	
Mother Nationality, *n* (%)	
Father Nationality, *n* (%)	
Child	171 (90.48)
Mother	162 (85.71)
Father	153 (80.95)
**Disease-specific variables**	
Diagnosis known within the first 3 months of life, *n* (%)	97 (51.32)
Stable disease course, *n* (%)	92 (48.68)
Number of misdiagnoses, *M* (*SD*), *Range*	0.43 (1.33), 0–7.5
Disease type, *n* (%)	
Q00–Q99: congenital malformations, deformations, and chromosomal abnormalities	105 (55.56)
D50–D89: diseases of the blood and blood-forming organs and certain disorders involving the immune mechanism	37 (19.58)
G00–G99: diseases of the nervous system	17 (8.99)
E00–E90: endocrine, nutritional, and metabolic diseases	30 (15.87)
Membership patient organization, *n* (%)	70 (37.03)
Healthcare utilization, *M* (*SD*), *Range*	5.71 (12.72), 0–100
Psychiatric diagnosis, *n* (%)	
Child	15 (7.94)
Parent/guardian	27 (14.29)
Counseling or psychotherapy, *n* (%)	
Child	19 (10.05)
Parent/guardian	39 (20.63)
**BCQ subscales**	
Expectations, *M* (*SD*), *Range*	75.11 (23.03), 10.71–100
Knowledge and Beliefs, *M* (*SD*), *Range*	81.08 (18.92), 18.75–100
Marginalization, *M* (*SD*), *Range*	82.65 (18.7), 20.45–100
Pragmatics, *M* (*SD*), *Range*	69.97 (18.18), 25–100
Skills, *M* (*SD*), *Range*	84.18 (13.64), 43.75–100
**PedsQL^TM^ (z-transformed)**	
Physical health, *M* (*SD*), *Range*	8.32 (3.52), 0–12.5
Psychosocial health, *M* (*SD*), *Range*	4.62 (1.99), 0–6.67

*Note.* BCQ=Barriers to Care Questionnaire; PedsQL^TM^=Pediatric Quality of Life Inventory; *M*=mean; *N*=sample size.

a 0 = special education school/not completed mandatory school education, completion of mandatory schooling (9 years), 1 = higher degree upper secondary school/secondary school and A-levels/technical school/seminar/university of applied sciences and University/ETH.

### Barriers to care classes and their compositions

**Figure 1.**
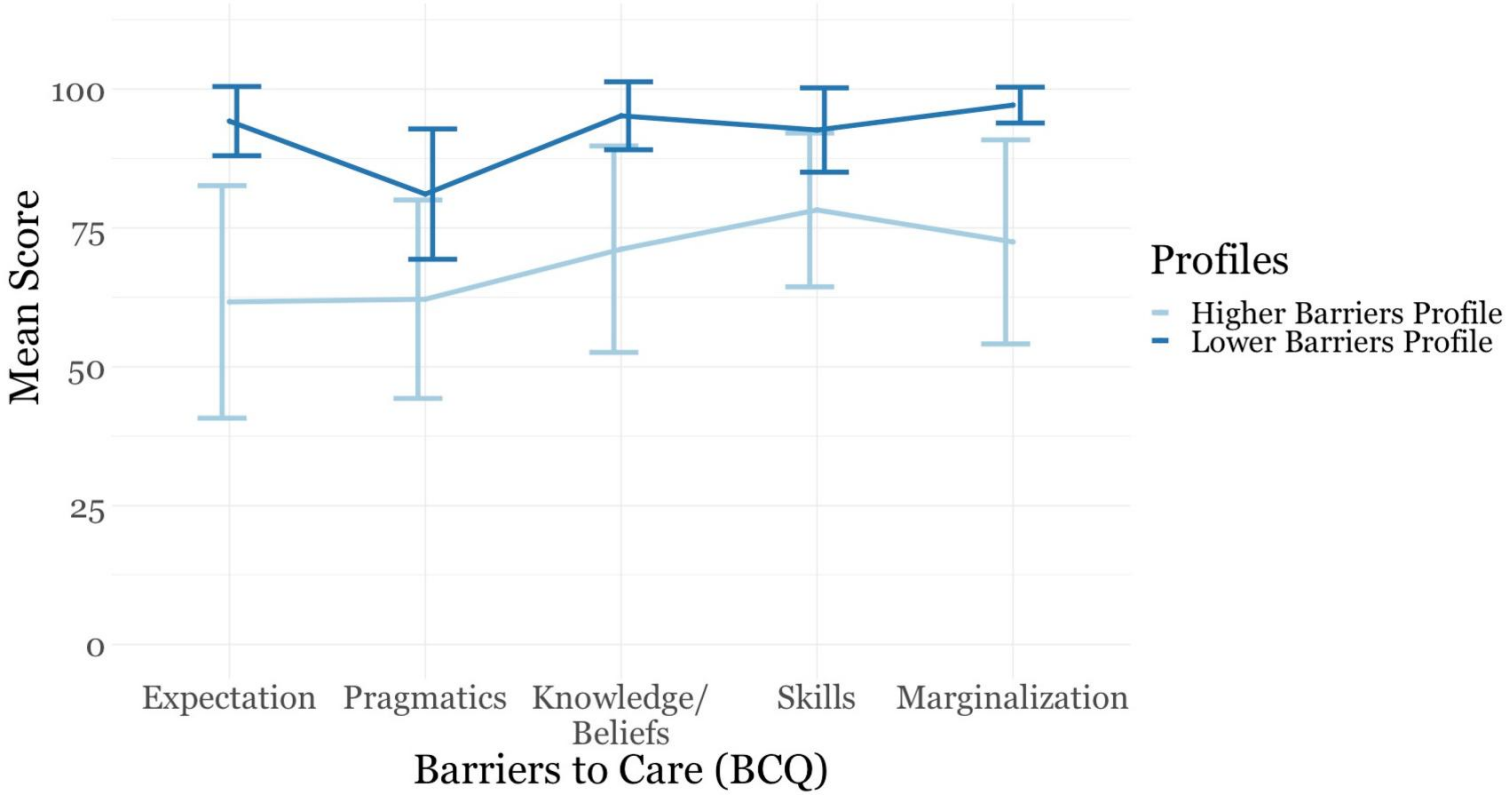
Mean subscale scores and confidence intervals for the Barriers to Care Questionnaire (BCQ) stratified by class.

Based on the statistical fit indexes, the present data revealed a two-class solution with varying variances and covariances as the best solution for LPA (Supplementary Table 3). As depicted in Figure 1, Class 1, comprising individuals showing more barriers across all subscales (*n *=* *111, 58.73%), was termed the “higher barriers class” (indicating lower BCQ scores across subscales). In contrast, Class 2, exhibited fewer barriers and higher BCQ scores across subscales (*n *=* *78, 41.27%), was termed the “lower barriers class.”

The higher and lower BCQ classes were compared across the five BCQ subscales revealing significant differences between classes (*p*<.001). The strongest effect was observed for the expectation subscale (*d *=* *1.97), followed by marginalization (*d *=* *1.73), knowledge and beliefs (*d *=* *1.62), skills (*d *=* *1.23), and pragmatics (*d *=* *1.21). All of these effect sizes were large.

When comparing BCQ subscales with one another in the context of the higher barriers class, significant differences (*p*<.01) emerged for nearly all subscales. However, the skills and marginalization subscales (*p*=.05) and the skills and knowledge and beliefs subscales (*p*=.52) did not. These results indicate that the highest scoring subscales did not significantly differ from one another. Conversely, the lowest-scoring expectations and pragmatics subscales did not differ (*p*=.88) but significantly differed from the three highest scoring subscales. The skills subscale had the highest mean score, followed by marginalization, knowledge and beliefs, pragmatics, and expectations (see Table 2). In contrast, when examining the BCQ subscales of the lower barriers class, the highest scores were found for marginalization, knowledge and beliefs, expectations, skills, and pragmatics.

**Table 2.** Model fit indices for model comparison.

Model	Entropy	*Log likelihood*	*p* (LMR LRT)	*p* (BLRT)	aBIC	AIC	BIC	CAIC	ICL
Sample used for analysis (*N *=* *189)									
Zero covariances—equal variances									
1-Class	–	−4,082.442	–	–	11,968.18	8,184.884	8,217.302	8,227.302	−8,217.302
2-Class	0.872	−3,894.996	<.001	<.01	11,605.78	7,821.992	7,873.860	7,889.860	−7,890.157
3-Class	0.886	−3,827.766	<.001	<.01	11,483.77	7,699.533	7,770.851	7,792.851	−7,793.403
4-Class	0.707	−3,827.346	<.001	.960	11,495.38	7,710.692	7,801.461	7,829.461	−7,891.929
5-Class	0.884	−3,787.523	<.001	<.01	11,428.20	7,643.045	7,753.264	7,787.264	−7,788.963
Varying covariances—varying variances									
1-Class	–	−3,834.377	–	–	11,492.80	7,708.753	7,773.588	7,793.588	−7,773.588
**2-Class**	**0.891**	**−3,670.103**	**<.001**	**<.01**	**11,207.81**	**7,422.206**	**7,555.118**	**7,596.118**	**−7,567.577**
Sample used for analysis, including excluded cases (*N *=* *196)									
Zero covariances—equal variances									
1-Class	–	−4,238.870	–	–	8,498.843	8,497.741	8,530.522	8,540.522	−8,530.522
2-Class	0.870	−4,047.868	<.001	<.01	8,129.499	8,127.736	8,180.185	8,196.185	−8,197.439
3-Class	0.882	−3,975.099	<.001	<.01	7,994.197	7,994.197	8,066.316	8,088.316	−8,090.807
4-Class	0.905	−3,948.579	<.001	.02	7,953.159	7,953.159	8,044.946	8,072.946	−8,070.376
5-Class	0.883	−3,932.983	<.001	<.01	7,933.966	7,933.966	8,045.422	8,079.422	−8,081.334
Varying covariances—varying variances									
1-Class	–	−3,979.546	–	–	8,001.296	7,999.092	8,064.654	8,084.654	−8,064.654
**2-Class**	**0.893**	**−3,804.205**	**<.001**	**<.01**	**7,694.929**	**7,690.410**	**7,824.813**	**7,865.813**	**−7,837.329**

*Note.* AIC=Akaike Information Criterion; BIC=Bayesian Information Criterion; aBIC=adjusted Bayesian Information Criterion; CAIC=Consistent Akaike Information Criterion; ICL=Integrated completed likelihood; LMR LRT=Lo–Mendell–Rubin Likelihood Ratio Test; BLRT=Bootstrapped Likelihood Ratio Test; *p *=* p*-value.

### Risk and protective factors between classes

Descriptive statistics comparing the two classes are presented in Table 3. The higher barriers class exhibited a significantly more misdiagnoses compared to the lower barriers class, as determined by a Mann–Whitney *U* test (*U* = 2,961). Chi-squared tests also revealed significant differences between class groups and disease-related factors. Specifically, there was a significant difference between class and diagnosis during the first 3 months of life (χ^2^(1)=9.575), with a higher number of individuals being diagnosed after 3 months of age in the higher barriers class. A similar pattern was observed for children having a psychiatric diagnosis (χ^2^(1)=4.069) and for children belonging to the ICD-10 subtype of congenital malformations, deformations, and chromosomal abnormalities (χ^2^(1)=4.128), that were more common in the higher barriers class.

**Table 3.** Descriptive statistics divided by class.

	Lower barriers class (*n *=* *78)	Higher barriers class (*n *=* *111)	*p*
**BCQ subscales**			
Expectations, *M* (*SD*), *Range*	94.23 (6.23), 82.14–100	61.68 (20.93), 10.71–100	<.001
Knowledge and Beliefs, *M* (*SD*), *Range*	95.19 (6.12), 81.25–100	71.17 (18.58), 18.75–100	<.001
Marginalization, *M* (*SD*), *Range*	97.12 (3.23), 88.64–100	72.48 (18.37), 20.45–100	<.001
Pragmatics, *M* (*SD*), *Range*	81.09 (11.73), 52.78–100	62.16 (17.87), 25–100	<.001
Skills, *M* (*SD*), *Range*	92.63 (7.59), 68.75–100	78.24 (13.84), 43.75–100	<.001
**Sociodemographic factors**			
Child age, *M* (*SD*), *Range*	7.24 (4.75), 1–17	7.79 (4.34), 1–17	.316
Child gender female, *n* (%)	27 (34.62)	50 (45.05)	.316
Education high^a^, *n* (%)			
Mother	70 (89.74)	95 (85.59)	.533
Father	66 (84.62)	90 (81.08)	.663
Medical cost coverage private, *n* (%)	10 (12.82)	20 (18.02)	.447
In a relationship or married, *n* (%)	74 (94.87)	74 (100)	.356
Swiss Nationality, *n* (%)			
Child	71 (91.03)	100 (90.09)	1.000
Mother	66 (84.62)	96 (86.48)	.880
Father	66 (84.62)	87 (78.38)	.375
**Disease-specific variables**			
Diagnosis known within the first 3 months of life, *n* (%)	51 (65.38)	46 (41.44)	<.01
Stable disease course, *n* (%)	31 (39.74)	61 (54.95)	.056
Number of misdiagnoses, *M* (*SD*), *Range*	0.19 (0.92), 0–7.5	0.61 (1.55), 0–7.5	<.01
Disease type, *n* (%)			
Congenital malformations, deformations, and chromosomal abnormalities	36 (46.15)	69 (62.16)	<.05
Diseases of the blood and blood-forming organs and certain disorders involving the immune mechanism	21 (26.92)	16 (14.41)	.051
Diseases of the nervous system	5 (6.41)	12 (10.81)	.434
Endocrine, nutritional, and metabolic diseases	16 (20.51)	14 (12.61)	.207
Membership patient organization, *n* (%)	28 (35.90)	42 (37.84)	.905
Healthcare utilization, *M* (*SD*), *Range*	8.15 (19.05), 0–100	3.99 (3.91), 0–20	.420
Psychiatric diagnosis, *n* (%)			
Child	2 (2.56)	13 (11.71)	<.05
Parent	12 (15.38)	15 (13.51)	.880
Counseling or psychotherapy, *n* (%)			
Child	7 (8.97)	7 (10.81)	.869
Parent	16 (20.51)	23 (20.72)	1.00

*Note. M*=mean; *n*=sample size; *p *=* p*-value; a= higher degree upper secondary school/secondary school and A-levels/technical school/seminar/university of applied sciences and University/ETH.

### Risk and protective factors associated with the higher barriers class

The results of Elastic Net analysis revealed that all sociodemographic factors held predictive value for class membership. However, none of the sociodemographic predictors significantly predicted class membership in the logistic regression analysis (Table 4). This suggests that while the model considered these factors relevant, their individual contributions were not strong enough to reach statistical significance.

**Table 4.** Sociodemographic and disease-specific predictors of the higher barriers to care class using a 2-class logistic regression model.

Effect	OR	B	*SE*	Wald	95% CI		*p*
					*LL*	*UU*	
Sociodemographic predictors							
Intercept	3.14	1.144	1.108	1.065	0.358	27.541	.302
Child age	1.02	0.015	0.036	0.179	0.946	1.089	.672
Child sex^a^	1.74	0.555	0.325	2.921	0.921	3.294	.087
Medical cost coverage^b^	1.43	0.360	0.447	0.650	0.597	3.442	.420
Relationship status^c^	0.62	−0.481	0.807	0.355	0.127	3.006	.551
Education^d^							
Mother	0.84	−0.174	0.458	0.145	0.342	2.062	.704
Father	0.57	−0.561	0.530	1.122	0.202	1.613	.290
Swiss nationality^e^							
Mother	0.64	−0.445	0.865	0.264	0.118	3.492	.607
Father	1.96	0.674	0.618	1.191	0.584	6.588	.275
Child	0.69	−0.367	0.515	0.507	0.252	1.901	.476
Custody^f^	1.60	0.473	1.083	0.191	0.192	13.406	.662
Disease-specific predictors							
Intercept	1.64	0.494	0.697	0.502	0.418	6.425	.478
Disease course^g^	2.27	0.818	0.376	4.719	1.084	4.735	<.05
Diagnosis not known before 3 months of age^h^	2.17	−0.781	0.398	3.851	0.210	0.999	<.05
Number of misdiagnoses	1.18	0.162	0.178	0.821	0.830	1.667	.365
Disease type^i^							
Congenital malformations, deformations, and chromosomal abnormalities	1.41	0.346	0.663	0.272	0.385	5.183	.602
Diseases of the blood and blood-forming organs and certain disorders involving the immune mechanism and diseases of the circulatory system	0.79	−0.242	0.734	0.109	0.186	3.309	.742
Endocrine, nutritional, and metabolic diseases	0.84	−0.175	0.773	0.051	0.185	3.819	.821
Membership patient organization^j^	0.83	−0.190	0.361	0.275	0.408	1.678	.600
Healthcare utilization (total number of medical visits)	0.94	−0.058	0.033	3.109	0.885	1.007	.078
Counseling or psychotherapy^k^							
Child	1.21	0.187	0.491	0.145	0.461	3.156	.704
Parent	0.72	−0.322	0.629	0.262	0.211	2.486	.609
Psychiatric diagnosis^l^							
Child	0.85	−0.157	0.545	0.084	0.294	2.487	.773
Parent	4.20	1.436	0.868	2.737	0.767	23.041	.098

*Note. N *=* *189. B=unstandardized regression coefficient; OR=odds ratio; CI=confidence interval; *LL*=lower limit; *UL*=upper limit; *p *=* p*-value.

a 0 = male, 1 = female.

b 0 = general insurance, self-coverage, and disability insurance, 1 = semiprivate and private insurance.

c 0 = not married nor in a relationship, 1 = married or in a relationship.

d 0 = special education school/not completed mandatory school education, completion of mandatory schooling (9 years), 1 = higher degree upper secondary school/secondary school and A-levels/technical school/seminar/university of applied sciences and University/ETH.

e 0 = no, 1 = yes.

f 0 = shared custody, 1 = sole custody.

g 0=stable, 1 = progressive, episodic, improving, and unknown.

h 0 = no, 1 = yes.

i 0 = no, 1 = yes.

j 0 = no, 1 = yes.

k 0 = no, 1 = yes.

l 0 = no, 1 = yes.

We then evaluated which disease-specific factors were predictive of membership in the higher barriers class. Except for the disease type labeled “diseases of the nervous system,” all predictors were retained in the logistic regression model. Subsequently, logistic regression analysis (using all predictors, see Table 4) revealed that a nonstable disease course was significantly associated with higher odds of belonging to the higher barriers class. Not being diagnosed within the first 3 months of life was predictive of lower odds of being in the higher barriers class.

### HRQoL in the higher barriers class

While controlling for child age, child sex, and the two significant predictors from the previous logistic regression analyses, children in the higher barriers to care class scored significantly lower on both the physical and psychosocial health summary score compared to those within the lower barriers class (see Table 5). From the control variables, only disease course reached significance, meaning that an unstable disease course contributed to this effect.

**Table 5.** Regression results predicting PedsQL summary scores.

	PedsQL *physical health summary score*						PedsQL *psychosocial health summary score*					
Effect	β	*SE*	95% CI		*p*	Fit	Β	*SE*	95% CI		*p*	Fit
			*LL*	*UU*					*LL*	*UU*		
(Intercept)	0.598	0.232	0.160	1.036	.011		0.598	1.900	−6.043	1.708	.257	
Barriers to care class^a^	−0.163	0.156	−0.306	−0.021	<.05		−0.431	0.480	−0.788	−0.074	<.05	
Disease course^b^	−0.408	0.154	−0.537	−0.280	<.001		−0.756	0.417	−1.044	−0.467	<.01	
Diagnosis known before 3 months of age^c^	0.020	0.154	−0.125	0.164	.800		−0.414	0.436	−0.775	−0.052	.050	
Child age	−0.015	0.017	−0.157	0.126	.836		0.336	0.140	0.012	0.660	.074	
Child sex^d^	−0.014	0.148	0.154	0.125	.844		0.061	0.372	−0.242	0.365	.715	
						*R* ^2^=.200 *p*<.001						*R* ^2^=0.113 *p*<.001
						*F(5, 155)* *=* *8.636*						*F(5, 155)* *=* *5.088*

*Note. N *=* *189. β=standardized regression coefficient; CI=confidence interval; *LL*=lower limit; *UL*=upper limit; *R*^2^=proportion of variance explained; *F *=* *F-statistic; *p *=* p*-value.

a 0 = lower barriers to care class, 1 = higher barriers to care class.

b 0 = stable, 1 = progressive, episodic, improving, and unknown.

c 0 = no, 1 = yes.

d 0 = male, 1 = female.

## Discussion

This study pursued three primary objectives. Firstly, we aimed to elucidate the multifaceted challenges and barriers to care in the context of rare pediatric conditions within the Swiss healthcare system using LPA. Secondly, we investigated potential predictors of barriers to care classes. In a final step, we investigated the relation between barriers to care and HRQoL.

### Latent profiles of barriers to care

One of the main findings of this study was the identification of two distinct barrier to care groups within the Swiss pediatric rare disease population. One group had comparably lower barriers to care, while the other faced higher barriers. The consistently large effect sizes between the classes across the BCQ subscales indicate that the classes are distinctly different and do not overlap. However, it is important to point out that there are no established cutoffs or minimal clinically meaningful differences for the BCQ.

While both classes showed distinct overall profiles of high or low BCQ scores, within the low-scoring class certain subscales showed comparatively higher or lower scores than others within the same class. It is important to note, however, that these scores remained lower than the corresponding subscale scores observed in the higher-scoring profile. In particular, the expectations subscale—which focuses on the expectation of poor quality of care, the fear that medical professionals make mistakes, and a lack of communication within the healthcare system, was the most endorsed barrier. This observation may be due to items relating to the lack of communication between the different actors in the healthcare system, as well as parents’ fears that doctors make mistakes and symptoms are overlooked (see Supplementary Table 4). Similarly, the most significant between-class difference was observed for the expectations subscale, which had the largest effect size. In the higher barrier class, the expectations subscale was the least endorsed barrier. In contrast, in the lower barriers class it was the most endorsed barrier indicating discrepant between-class effect sizes. This difference underscores the importance of the expectations subscale and its role in differentiating the two classes.

In contrast, the marginalization subscale did not present a significant barrier for the higher barriers class. This observation suggests that parents/guardians generally feel respected and treated fairly by healthcare providers. However, this absence of perceived marginalization stands in contrast to the notable challenges families face in feeling heard and understood. As the primary barriers relate to concerns pertaining to the expectations subscale which focuses on quality of care, provider expertise, and communication. While a lack of medical expertise has traditionally been viewed as a potential barrier to early diagnosis and treatment of rare pediatric conditions, evidence suggests that inadequate communication between parents and physicians (Kohlschütter & van den Bussche, 2019) and the lack of person-centered care (Kocher et al., 2021) may also play a pertinent role. This underscores the importance of shared decision-making to promote trust and autonomy in the treatment process. Parental trust can be particularly fragile when communication is poor or ineffective. Parents of children with rare diseases often report knowing more about their child’s condition than physicians, which can undermine their trust in healthcare professionals when they receive inaccurate information (Kocher et al., 2021). Families affected by rare diseases place a high value on healthcare providers actively listening to their concerns and requests, underscoring the critical role of mutual trust in healthcare (Mazzella et al., 2021). Future interventions could benefit from focusing on reducing communication barriers and fostering a more collaborative relationship between parents and providers, such as shared decision-making (Kohlschütter & van den Bussche, 2019).

When comparing BCQ subscales in the higher barriers profile, we observed significant differences in the expectations and pragmatics subscales (compared to the other three subscales). Notably, both barriers were the most endorsed barriers and were not significantly different from each other. Within the pragmatics subscale, items related to parents’ time constraints (e.g., long waits for appointments and in the waiting room), family and caregiving responsibilities, and financial barriers (e.g., cost of healthcare and need to take time off from work) were the driving factors for this effect (see Supplementary Table 3). These findings are consistent with previous research showing that parents of affected children face a variety of family and work challenges to ensure regular access to healthcare for their child (Pelentsov et al., 2015; Zurynski et al., 2017). Additionally, two recent studies have shown that out-of-pocket costs for families dealing with rare diseases are significant (Deverell et al., 2022; Teutsch et al., 2023). This situation exacerbates healthcare access inequities for affected families, putting them at risk of financial hardship, amplified by a loss of income resulting from increased caregiving responsibilities (Pelentsov et al., 2015). Future initiatives should prioritize interventions aimed at minimizing wait times and enhancing financial and caregiver support for affected families, thereby alleviating barriers to care. One effective approach is the implementation of telemedicine, which has demonstrated effectiveness in reducing the time to specialist consultation (Pfeil et al., 2023).

### Group differences regarding barriers classes

When comparing the two classes, significant differences emerged related to the timing of diagnosis (i.e., during the first 3 months of life), disease type, number of misdiagnoses, and presence of a child with a psychiatric diagnosis. Notably, children diagnosed with the ICD-10 subtype of “congenital malformations, deformations, and chromosomal abnormalities” were more prevalent in the higher barriers class. This observation may be linked to the increased medical complexity often associated with this specific diagnostic group. Children in this category typically require multidisciplinary care and care coordination adding to the complexity of interactions with the healthcare system (Kalbfell et al., 2023). A qualitative study of parents of children diagnosed with complex vascular malformations highlighted that access to healthcare required the involvement of multidisciplinary teams. Parents had to complete multiple forms and gather medical information from multiple sources while seeing physicians in different locations. This process was found to be very stressful for the parents (Sisk et al., 2022). One potential intervention could involve the utilization of patient navigators and case managers, proven to be valuable in assisting individuals in navigating healthcare (Kelly et al., 2019).

The higher frequency of misdiagnosis in the higher barrier class is consistent with existing literature highlighting the strong association between the frequency of misdiagnosis and health-related factors, particularly access to disease-related information (Dong et al., 2020). Research suggests that the occurrence of misdiagnosis is closely related to low disease awareness and limited knowledge among health professionals (Vandeborne et al., 2019). Such findings are further supported by consistently low scores in the knowledge and beliefs and expectation subscales in both classes. Accordingly, prior misdiagnosis(es) may potentially lead parents/guardians to have a more critical and cautious approach when weighing medical advice. Past experiences may include pitfalls and uncertainties in relation to healthcare-related decisions, that shape parental/guardian perceptions and attitudes toward healthcare professionals.

Of note, the class with higher barriers also reported a higher incidence of psychiatric diagnoses. When psychiatric comorbidities are present, parents face the additional challenge of managing their child’s psychiatric care in conjunction with pediatric primary care, that may compound barriers to care for affected families (Parekh & Barton, 2010). Children and adolescents with rare diseases exhibit increased vulnerability to a range of externalizing and internalizing psychiatric symptoms, as well as developmental disorders (e.g., autism spectrum disorder) (Toly et al., 2012). The presence of comorbid mental disorders alongside chronic illness has been associated with worsening disease course and contributes to decreased quality of life (Müller-Tasch et al., 2007). Future research should prioritize examining the complex relationship between psychiatric comorbidities, chronic illness, and barriers to care to improve the overall well-being of affected children and their families.

### The predictive role of disease course and early diagnosis regarding barriers to care

We identified two disease-related predictors significantly associated with membership in the higher barriers class: A nonstable disease course and diagnosis during the first 3 months of life. Previous research in the realm of pediatric and adult rare diseases has consistently shown that a nonstable disease course is linked to higher anxiety levels. Anxiety may arise from the stressful experience of living with an unpredictable disease (Bogart et al., 2022). In contrast, stable rare diseases are characterized by reduced uncertainty and increased perceived manageability, not only for patients but also for healthcare providers (Bogart & Irvin, 2017).

We also found that diagnosis after the first 3 months of life was associated with higher odds of belonging to a higher barrier class. Early diagnosis of rare diseases has had a positive impact, mitigating impaired HRQoL of affected individuals and reduced family stress, anxiety, and frustration. Moreover, early diagnosis serves to shorten diagnostic delays and the arduous diagnostic odyssey that families dealing with rare diseases often experience (Bogart & Irvin, 2017; Zurynski et al., 2017). Furthermore, early diagnosis has been shown to facilitate timely interventions and more effective disease management. Early interventions help parents navigate the healthcare system more easily and reduce the likelihood that symptoms will become more complex and severe as the disease progresses (Kole & Faurisson, 2010). One strategy to improve early diagnosis is newborn screening, which is already offered in a number of countries for several conditions. A recent study involving parents whose children were diagnosed with metachromatic leukodystrophy found that newborn screening was highly beneficial because it enabled early diagnosis and intervention, potentially altering the course of the disease (Morton et al., 2022).

### Barriers to care and association with HRQoL

Being in the higher barriers class significantly predicted lower HRQoL scores on both composite physical and psychosocial scores. Our findings align with the theoretical model proposed by Seid et al. (2004) and previous work in the field of pediatric chronic diseases (Molzon et al., 2018). The link between lower barriers to care and child health outcomes remains poorly understood and has received scant attention. However, there is growing evidence suggesting potential roles of moderating and mediating variables—such as illness uncertainty and caregiver distress. Illness uncertainty is a common experience for families dealing with rare diseases (Pelentsov et al., 2015). Parental mental health has been identified as a potential mediator in the relationship between barriers to care and child HRQoL (Molzon et al., 2018). Future research should aim to unravel the complexities of the observed relationship, considering potential mediators and moderators to better understand how barriers to care impact child health in the context of rare and chronic diseases.

### Strengths and limitations

A strength of this study is the rather sizeable sample of families affected by rare diseases and the heterogeneity of the diseases included. This clinical heterogeneity of the present sample may of course also be a weakness, as it may mask potential commonalities within specific diseases; however, affected individuals report many common experiences (Uhlenbusch et al., 2019b). In addition, we identified distinct classes that can inform targeted interventions—consistent with the principles of precision healthcare. The cross-sectional nature of this study limits our ability to draw any conclusions about the potential stability of barriers over time. Thus, future research should focus on examining whether these risk and protective factors precede more barriers to care or whether barriers to care contribute to the development of additional risk factors. Similarly, it is unclear whether poorer physical and mental health outcomes result from increased barriers to care or whether it, in turn, exacerbates barriers. Longitudinal studies are essential to explore these questions further and assess the dynamics of these barriers over time.

It is important to note that the structure and financing of healthcare systems vary widely between countries. This study was conducted in Switzerland, a country known for its generally high-quality healthcare system with primary care that contributes to perceived health equity (Jenni & Sennhauser, 2016). However, it is possible that outcomes could be different in health systems with less equitable access. Such differences in health systems could also lead to different health outcomes that may be more affected by barriers to care.

Furthermore, this study used LPA to create different groups reflecting different levels of barriers to care because no existing cutoffs were available. Accordingly, it remains unclear whether what we consider “higher barriers to care” in this study would be considered “high” in other national and international samples, regardless of whether they are rare or chronic diseases. Moreover, it is unclear whether these higher barriers are clinically meaningful. Nonetheless, our results suggest that the lower barriers class faces more severe HRQoL impairments, highlighting the challenges they face. Future research should aim to examine BCQ thresholds and their associations with treatment outcomes related to rare diseases in different health systems and patient populations.

### Clinical relevance and implications

This study highlights the importance of comprehensively assessing barriers to care related to rare diseases in pediatrics. Our results suggest distinct classes characterized by varying degrees of barriers to care, including a lower- and higher-barriers class. Significant differences among these classes underscore the critical influence of early detection, disease course, disease type, misdiagnosis, and psychiatric comorbidity. Insight into these factors is essential for tailoring interventions and reducing barriers to care for families affected by rare diseases. Notably, within the higher barriers class, two specific BCQ subscales were the most endorsed barriers: Expectations and pragmatics. These subscales highlight parents’ concerns about the quality of medical care, communication, waiting times, family obligations, and financial constraints. One potential intervention could be the active prompting of parents by health professionals, encouraging them to actively participate in the decision-making process (Hubner et al., 2018). As another potential intervention to address the issue of parent–provider trust, we propose the importance of role-playing as an exercise to train communication skills at the provider, parent, and child levels (Kodjebacheva et al., 2016). To improve access to medical care for rare pediatric conditions, it is critical to comprehensively address these barriers and incorporate them into clinical guidelines. Such guidelines could recommend the routine screening and monitoring of barriers to care and specific interventions. Furthermore, being in the higher barriers class was a reliable predictor of significantly lower PedsQL scores for both physical and psychosocial total scores. This association is consistent with the theoretical model of Seid et al. (2004) and is consistent with previous research on pediatric chronic conditions (Molzon et al., 2018), which confirms the association between barriers to care and health outcomes in children. In summary, targeted interventions are needed to improve access to healthcare and to address challenges related to expectations and pragmatics.

## Supplementary Material

jsae076_Supplementary_Data

## Data Availability

Data are available on request.
