# Supplementary material for: Latent profiles and predictors of barriers to care in Swiss children and adolescents with rare diseases
Source: J Pediatr Psychol. 2024 Sep 24;49(11):827–39. doi: 10.1093/jpepsy/jsae076 (PMC11812576; doi:10.1093/jpepsy/jsae076)
Supplement: jsae076_Supplementary_Data [file jsae076_supplementary_data.zip › jsae076_Supplementary_Data/jpepsy-2023-0256-File008.docx]

**Supplemental Table 1.**

*Disease type according to ICD-11 categorization (N = 189)*

|  | | *Sample (N = 189)* |
| --- | --- | --- |
| Disease type, *n* (%) | |  |
| Diseases of the blood or blood-forming organs | 7 (5.82) |  |
| Diseases of the digestive system | 1 (0.53) |  |
| Diseases of the genitourinary system | 1 (0.53) |  |
| Diseases of the immune system | 18 (9.52) |  |
| Diseases of the nervous system | 11(6.34) |  |
| Diseases of the respiratory system | 1 (0.53) |  |
| Diseases of the skin | 2 (1.06) |  |
| Developmental anomalies | 102 (53.97) |  |
| Endocrine, nutritional or metabolic diseases | 29 (15.34) |  |
| Only in the ICD-10 | 12 |  |
