# Supplementary material for: Latent profiles and predictors of barriers to care in Swiss children and adolescents with rare diseases
Source: J Pediatr Psychol. 2024 Sep 24;49(11):827–39. doi: 10.1093/jpepsy/jsae076 (PMC11812576; doi:10.1093/jpepsy/jsae076)
Supplement: jsae076_Supplementary_Data [file jsae076_supplementary_data.zip › jsae076_Supplementary_Data/jpepsy-2023-0256-File009.docx]

**Supplemental Table 2.**

*Excluded disease types according to ICD-10 categorization*

| Disease type, *n* | |  |
| --- | --- | --- |
| Diseases of the digestive system | 1 |  |
| Diseases of the genitourinary system | 1 |  |
| Diseases of the musculoskeletal and connective tissue | 1 |  |
| Neoplasms | 4 |  |
