# Supplementary material for: Latent profiles and predictors of barriers to care in Swiss children and adolescents with rare diseases
Source: J Pediatr Psychol. 2024 Sep 24;49(11):827–39. doi: 10.1093/jpepsy/jsae076 (PMC11812576; doi:10.1093/jpepsy/jsae076)
Supplement: jsae076_Supplementary_Data [file jsae076_supplementary_data.zip › jsae076_Supplementary_Data/jpepsy-2023-0256-File010.docx]

**Supplemental Table 3.**

*Descriptive statistics of items divided by class*

| **Item** | ***M* (*SD*)** | | |
| --- | --- | --- | --- |
|  | Lower barriers class  (*n* = 78) | Higher barriers class  (*n* = 111) | Total sample  (*N* = 189) |
| **Knowledge & Beliefs** |  |  |  |
| Disagreeing with the doctor’s orders. | 94 (11) | 69 (25) | 79 (24) |
| Doctors not believing in home or traditional remedies. | 96 (9) | 80 (26) | 86 (22) |
| Doctors giving you instructions that seem wrong. | 96 (9) | 67 (28) | 79 (26) |
| Doctors or nurses that have different ideas about health than you do. | 95 (12) | 69 (26) | 80 (24) |
| **Pragmatics** |  |  |  |
| Getting to the doctor’s office. | 85 (15) | 76 (27) | 80 (23) |
| Getting hold of the doctor’s office or clinic by phone. | 87 (16) | 73 (27) | 79 (24) |
| Having to wait too many days for an appointment. | 85 (20) | 56 (33) | 68 (32) |
| Getting care after hours or on the weekends. | 86 (20) | 70 (30) | 76 (27) |
| Having to take care of household responsibilities. | 81 (24) | 61 (30) | 70 (29) |
| Having to take time off work. | 72 (28) | 51 (34) | 60 (33) |
| Having to wait too long in the waiting room. | 79 (25) | 56 (33) | 66 (32) |
| Meeting the needs of other family members. | 71 (25) | 53 (29) | 60 (29) |
| The cost of health care. | 85 (15) | 62 (30) | 71 (28) |
| **Skills** |  |  |  |
| Knowing how to make the health care system work for you. | 84 (20) | 63 (28) | 71 (27) |
| Doctors or nurses not fluent in your language. | 83 (19) | 88 (23) | 92 (19) |
| Doctors or nurses who speak in a way that is too technical or medical. | 98 (7) | 82 (19) | 87 (18) |
| Getting referrals to specialists. | 92 (14) | 86 (22) | 91 (18) |
| Understanding doctor’s orders | 98 (7) | 90 (18) | 93 (15) |
| Having enough information about how the health care system works. | 97 (9) | 77 (23) | 84 (21) |
| Needing to be more ‘‘savvy’’ or knowledgeable about getting health care. | 93 (13) | 75 (22) | 82 (21) |
| Getting enough help with paperwork or forms. | 93 (15) | 64 (29) | 74 (28) |
| **Expectations** |  |  |  |
| Offices and staff that are not child-friendly. | 87 (19) | 76 (25) | 84 (23) |
| Mistakes made by doctors or nurses. | 96 (10) | 61 (30) | 74 (29) |
| Worrying that doctors and nurses will not do what is right for your child. | 94 (12) | 64 (26) | 76 (26) |
| Doctors treating the symptom without finding out the cause of the illness. | 94 (11) | 58 (34) | 73 (33) |
| Getting a thorough examination. | 95 (12) | 67 (31) | 79 (28) |
| Lack of communication between my child’s doctor and others in the health care system. | 97 (9) | 56 (31) | 72 (31) |
| Lack of communication between different parts of the health care system. | 93 (14) | 51 (32) | 67 (33) |
| **Marginalization** |  |  |  |
| Feeling like doctors are trying to give as little service as possible. | 89 (18) | 67 (30) | 80 (28) |
| Feeling like the health care system is trying to give as little service as possible. | 98 (6) | 58 (33) | 72 (33) |
| Impatient doctors. | 91 (21) | 74 (29) | 84 (26) |
| Intimidating doctors. | 99.3590 (3.9771) | 78 (29) | 87 (25) |
| Rude office staff. | 99.6795 (2.8307) | 73 (29) | 83 (26) |
| Uncaring office staff. | 97 (9) | 70 (30) | 81 (27) |
| Getting the doctor to listen to you. | 97 (8) | 71 (27) | 82 (25) |
| Getting your questions answered. | 98.72 (5.55) | 67 (29) | 79 (27) |
| Not knowing what to expect from one visit to the next. | 96 (9) | 70 (25) | 79 (24) |
| Being judged on your appearance, your ancestry, or your accent. | 92 (14) | 90 (23) | 94 (18) |
| Doctors rushing you and your child through the visit. | 100.0000 (0.0000) | 79 (26) | 87 (23) |

*Note.* *M* = mean. *n* = sample size. *SD* = standard deviation.
